# Different Biomarker Kinetics in Critically Ill Patients with High Lactate Levels

**DOI:** 10.3390/diagnostics10070454

**Published:** 2020-07-04

**Authors:** Ryo Matsuura, Yohei Komaru, Yoshihisa Miyamoto, Teruhiko Yoshida, Kohei Yoshimoto, Yoshifumi Hamasaki, Masaomi Nangaku, Kent Doi

**Affiliations:** 1 Department of Nephrology and Endocrinology, The University of Tokyo Hospital, 7-3-1 Hongo, Bunkyo-ku, Tokyo 113-8655, Japan; rimatsuura-tky@umin.ac.jp (R.M.); komaru-tky@umin.ac.jp (Y.K.); ymiyamoto70@gmail.com (Y.M.); yoshidateruhiko@gmail.com (T.Y.); yhamasaki-tky@umin.ac.jp (Y.H.); mnangaku-tky@umin.ac.jp (M.N.); 2 Department of Acute Medicine, University Hospital, The University of Tokyo Hospital, 7-3-1 Hongo, Bunkyo-ku, Tokyo 113-8655, Japan; yoshimotok-icu@h.u-tokyo.ac.jp

**Keywords:** lactate, interleukin-6, high-mobility group box 1, neutrophil gelatinase-associated lipocalin

## Abstract

We evaluated the association of the kinetics of interleukin-6 (IL-6), neutrophil gelatinase-associated lipocalin (NGAL), and high-mobility group box 1 (HMGB1) with intensive care unit (ICU) mortality in critically ill patients with hyperlactatemia. This proof-of-concept study was conducted with prospectively enrolled patients admitted to a medical/surgical ICU with hyperlactatemia (lactate levels >4 mmol/L). Blood lactate, IL-6, NGAL, and HMGB1 were measured every 2 h until 6 h post ICU admission. The primary outcome was ICU mortality. Of thirty patients in this study, 14 patients (47%) had sepsis, and the ICU mortality was 47%. IL-6 and NGAL levels were significantly higher in septic patients than in non-septic patients. On kinetic analysis, the lactate levels were significantly decreased in survivors, and the NGAL levels were significantly increased in non-survivors. Among septic patients, a decline in IL-6 levels were observed in survivors. The HMGB1 levels were unchanged in survivors and non-survivors regardless of sepsis complication. Non-septic patients with higher reduction rate of lactate and HMGB1 had the lowest mortality than the others. ICU patients exhibited different kinetic patterns in lactate, NGAL, and IL-6, but HMGB1 did not seem to change over the 6-h duration. Further studies are necessary to evaluate the efficacy of the combination of the inflammatory biomarkers with lactate.

## 1. Introduction

The mortality rate of critically ill patients treated in intensive care units (ICUs) is approximately 10–30% [1,2]. Identifying treatment responders and accurately predicting mortality will help in determining risk stratification. However, ICU includes heterogeneous populations of critically ill patients, including those post-surgery and those with cardiovascular disease, trauma, and sepsis. This presents a challenge in using biomarkers to predict outcomes [3,4]. Furthermore, serial measurements may provide more information in a heterogeneous patient population rather than single-point measurements. Some prospective observational studies showed that decreased lactate levels at 6 h post ICU admission were associated with decreased hospital mortality in patients with sepsis [5,6]. A multicenter randomized controlled trial also confirmed that lactate-guided therapy with reduced lactate levels by ≥20% in <8 h led to reduced mortality [7]. A meta-analysis showed that lactate clearance is associated with mortality in critically ill patients, including those with sepsis [8], suggesting that lactate kinetics may be used as a biomarker for sepsis treatment.

Inflammatory biomarkers, such as interleukin-6 (IL-6), neutrophil gelatinase-associated lipocalin (NGAL), and high-mobility group box 1 (HMGB1), have been effective at differentiating sepsis, predicting prognosis, and monitoring treatment in patients in the ICU. For instance, IL-6 has been shown to be associated with mortality, and its kinetics is an indicator of therapeutic response in patients with sepsis [9,10]. NGAL, known as an acute kidney injury (AKI) biomarker, can differentiate patients with sepsis from those without sepsis and is a significant prognostic factor for mortality [11]. HMGB1 is associated with illness severity and is persistently elevated in the non-surviving population of patients with sepsis [12,13].

However, it remains unclear whether serial measurements of these inflammatory biomarkers in short duration will provide more information than single measurements. Kinetic analysis in hours may be expected to help early recognition of treatment response. Of note, recent sepsis guidelines suggested several sepsis bundles within 3 and 6 h [14]. It is also necessary to evaluate if kinetics of these inflammatory biomarkers will be better than lactate kinetics. It is possible that different biomarkers will show different temporal patterns during initial treatment. Moreover, while many studies described moderate hyperlactatemia [15,16], there are limited data on patients with high lactate level >4 mmol, which increases ICU admission and mortality of >50% [15]. High initial values will be also useful for addressing the significance of clearance. This proof-of-concept study aimed to evaluate these biomarker kinetics in critically ill patients with hyperlactatemia (lactate levels >4 mmol/L) and clarify the utility of serial measurements.

## 2. Materials and Methods

### 2.1. Study Protocol

The study protocol, which was approved by the University of Tokyo institutional review board (#2810), adhered to the Declaration of Helsinki. Written informed consent was obtained on ICU admission. The prospectively enrolled patients were admitted to the medical/surgical ICU in the University of Tokyo Hospital during April 2015 through May 2016 and had hyperlactatemia (lactate levels >4 mmol/L). The following clinical variables were collected during ICU and hospital stay: age, sex, weight, causes of ICU admission, Acute Physiology and Chronic Health Evaluation (APACHE) II score, length of ICU stay, and length of hospital stay. AKI was defined as an increase in serum creatinine levels by 0.3 mg/dL within 48 h or an increase in serum creatinine to 1.5 times of the baseline according to the Kidney Disease Improving Global Outcomes (KDIGO) guideline [17]. Presence of sepsis was diagnosed according to the International Society of Critical Care Medicine (SCCM)/ European Society of Intensive Care Medicine (ESICM)/American College of Chest Physicians (ACCP)/American Thoracic Society (ATS)/Surgical Infection Society (SIS) definition [18]. Outcome was defined as mortality in the ICU.

### 2.2. Measurement of Lactate, IL-6, NGAL, and HMGB1 Levels

Plasma samples were collected every 2 h until 6 h post ICU admission and were frozen at −80 °C within 1 h of collection. Lactate levels were measured by the analysis of arterial blood gas (ABL800 FLEX, Radiometer, Denmark). Plasma IL-6 levels were measured by a two-step sandwich method using the Human IL-6 RAYFAST system (Toray, Tokyo, Japan) [19]. Plasma NGAL levels were measured using the Triage NGAL device (Alere Medical, San Diego, CA, USA). Plasma HMGB1 levels were determined using an enzyme-linked immunosorbent assay kit (Fuso, Osaka, Japan), according to the manufacturer’s protocol. Reduction rates of each biomarker were calculated as the ratio of the value at each time compared with that at baseline (0 h).

### 2.3. Statistical Analysis

Continuous data were presented as median (interquartile) and categorical data frequencies (percentage). Continuous variables were compared between groups or between two different days in the same group using Wilcoxon rank-sum tests or Friedman’s test. The Bonferroni correction was used for multiple comparison. Reduction rates of each biomarker for predicting mortality were evaluated using a receiver operating characteristic (ROC) curve analysis. The optimal cutoff values were acquired using the Youden index (sensitivity + specificity − 1), which is a common summary measure of the ROC curve representing the maximum potential effectiveness of a marker [20]. When the effect of the combination with lactate and other biomarkers was analyzed, the proportion of ICU mortality between groups was compared using Fisher’s exact test. The effect of the combination with lactate and other biomarkers was also evaluated using net reclassification index and integrated discrimination improvement [21,22].

## 3. Results

### 3.1. Baseline Characteristics

Thirty patients were analyzed in this study. Twenty-one patients (70%) were admitted for medical conditions, and 14 patients (47%) were complicated with sepsis. All patients with sepsis were determined as septic shock according to the International SCCM/ESICM/ACCP/ATS/SIS definition [18]. A total of 6 of 14 patients had positive blood cultures. Infection sites were mainly gastrointestinal and lung, and infecting organisms consisted of Gram-positive cocci, Gram-positive rod, and Gram-negative rod (Table 1). Patients with sepsis received the standard care and were managed according to the best practice as recommended by the Surviving Sepsis Campaign guidelines 2012 [23]. Majority of patients had AKI (83%) and respiratory failure that needed mechanical ventilation (73%). No patients had factors affecting lactate level, such as metformin use and liver failure. The mortality rate of these 30 patients in the ICU was 47%.

### 3.2. Sepsis and Inflammatory Biomarkers at ICU Admission

**Figure 1:**
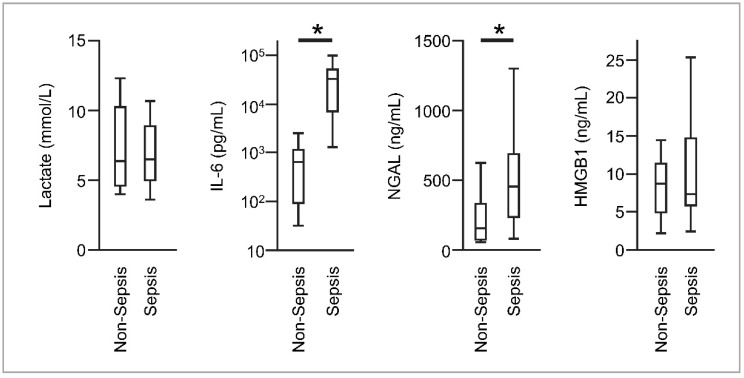
Boxplots of lactate, interleukin-6 (IL-6), neutrophil gelatinase-associated lipocalin (NGAL), and high-mobility group box 1 (HMGB1) levels at intensive care unit (ICU) admission in patients with and without sepsis. The error bars show the total range. * *p* < 0.01.

Median blood lactate, NGAL, IL-6, and HMGB1 levels in all patients at ICU admission were 6.4 (interquartile range [IQR], 4.8–9.5) mmol/L, 285 (IQR, 116–518) ng/mL, 1444 (IQR, 516–28,103) pg/mL, and 8.2 (IQR, 5.8–12.3) ng/mL, respectively. The NGAL and IL-6 levels were significantly higher in patients with sepsis than in those without sepsis (NGAL: 454 (IQR, 240–690) vs. 155 (IQR, 74–331) ng/mL; IL-6: 32325 (IQR, 7029–51,380) vs. 642 (IQR, 93–1141) pg/mL). There was no significant difference in blood lactate and HMGB1 levels between the groups (Figure 1).

### 3.3. Biomarker Kinetics and ICU Mortality

**Figure 2:**
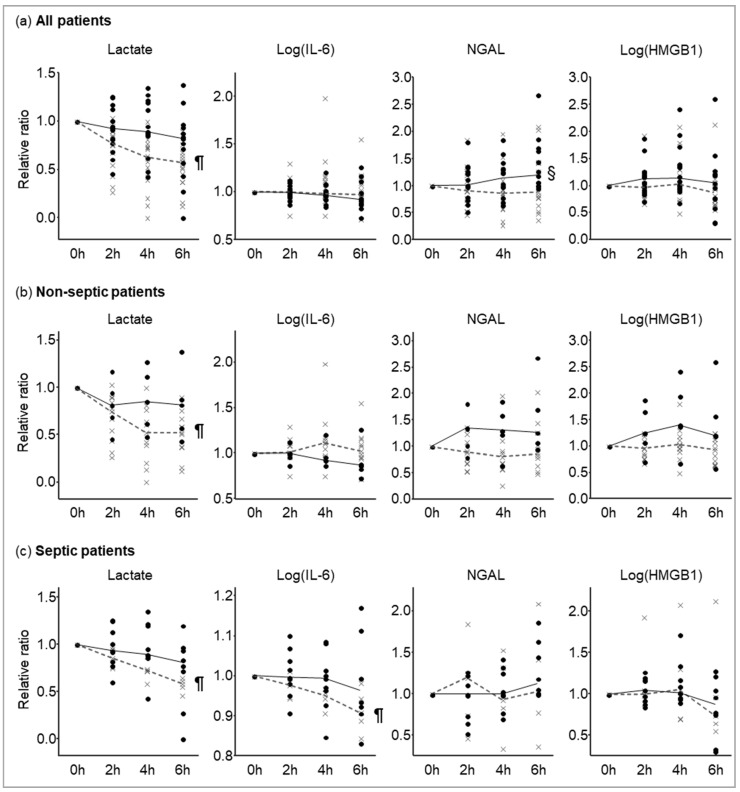
Lactate, IL-6, NGAL, and HMGB1 kinetics in (**a**) all patients, (**b**) patients without sepsis, and (**c**) patients with sepsis. Solid lines and black circles indicate non-survivors; dashed line and gray x-marks indicate ICU survivors. Solid and dashed lines connect the median at each time. ¶: *p* < 0.05 compared with 0 h in survivors. §: *p* < 0.05 compared with 0 h in non-survivors.

Inflammatory biomarker reduction rates among ICU survivors and non-survivors are shown in Figure 2. Friedman’s test revealed that blood lactate levels significantly declined after ICU admission for ICU survivors but not for non-survivors, while other biomarkers did not significantly change. By Wilcoxon’s rank sum test, the NGAL levels at 6 h ICU admission were significantly higher than those at admission in non-survivors, whereas no significant changes in NGAL levels were observed in survivors. Similarly, NGAL kinetics was observed in patients with and without sepsis; however, there was no statistically significant difference in the NGAL kinetics possibly because of a small sample size. A significant decline in IL-6 levels at 6 h post ICU admission was observed in the ICU survivors with sepsis, but not in those without sepsis. HMGB1 levels were unchanged in all patients, even when the patients were divided into those with and without sepsis.

### 3.4. Combination of Biomarkers and ICU Mortality

**Figure 3:**
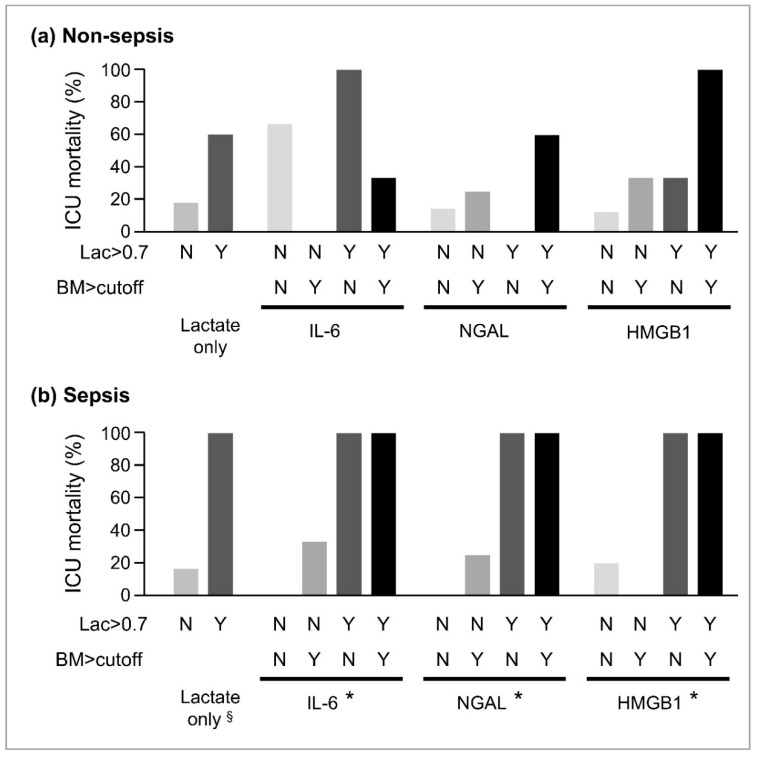
Combination with lactate and other biomarkers.

We analyzed whether the combination of lactate and other biomarkers would improve the capacity to predict the outcome. First, receiver operating characteristic analysis was conducted on each biomarker reduction rate for 6 h (Table 2). The cutoff values of the relative changes for each biomarker were determined by Youden’s index. The lactate clearance cutoff value alone could not predict mortality in the non-septic patients, while the septic patients were clearly discriminated regarding mortality by the lactate clearance cutoff value of 0.7 (Figure 3a). When the non-septic patients showed lower clearance of lactate and HMGB1 based on these cutoff values, they had the highest mortality compared with the others (*p* = 0.09) (Figure 3b). Therefore, the addition of HMGB1 to lactate may help stratify the risk more accurately. However, analysis of net reclassification improvement and integrated discrimination improvement could not provide the efficacy of the combination of biomarkers with lactate (Table S1).

Receiver operating characteristic analysis was conducted on each biomarker reduction rate for 6 h. Each biomarker reduction rate is the same as in Figure 2. Cutoffs are the values of relative ratio at 6 h compared with those at 0 h. CI, confidence interval; IL-6, interleukin-6; HMGB1, high morbidity group box 1; NGAL. neutrophil gelatinase-associated lipocalin.

The effect of the combination with lactate and each other biomarker (BM) was analyzed for (a) non-septic and (b) septic patients. The enrolled patients were divided to four groups according to the cutoff value of 6 h/0 h ratio determined by the ROC analysis (Table 2). Y denotes elevation of the biomarker to more than the cutoff values, and N denotes otherwise. §: *p* < 0.05 in comparison between the N group and the Y group. *: *p* < 0.05 in comparison among the N/N group, N/Y or Y/N group, and Y/Y group.

## 4. Discussion

This study described different early inflammatory biomarker kinetics in patients with severe illness. Blood lactate was rapidly cleared in ICU survivors; this result was consistent with that observed in previous reports. In addition, we evaluated the association of inflammatory marker kinetics with the prognosis of critically ill patients. The findings of our study are as follows: (a) early IL-6 reduction was associated with ICU survival in patients with sepsis; (b) NGAL levels increased in non-survivors; (c) HMGB1 levels were unchanged in survivors and non-survivors at least within 6 h post ICU admission, but the combination of HMGB1 with lactate may stratify the risk of mortality.

Single measurement of biomarker has a limitation. The biomarker level at one point is affected by production and elimination. Given these limitations, serial measurements are more informative as an outcome predictor than a single measurement. By contrast, the kinetics of prognostic markers will enable to monitor whether currently ongoing treatment is improving the patients’ condition or not. Lactate is one of the biomarkers of which serial measurements are predictive of prognosis. Furthermore, this study found that the inflammatory markers—IL-6, NGAL, HMGB1—as well as lactate, have the potential to predict ICU mortality. Although all the patients in this study were treated by standard therapeutic management against septic shock, this monitoring may help us identify the non-responder to the standard treatment and to be useful for clinical trials that will evaluate novel treatment for sepsis especially for enrollment.

IL-6 has a prognostic role in differentiating patients with sepsis from those without sepsis [24]. In this study, IL-6 levels in patients with sepsis were significantly higher than those in patients without sepsis. Moreover, sepsis survivors showed a significant reduction in IL-6 levels at 6 h post ICU admission from the baseline levels. This result suggested that IL-6 kinetics in short duration of 6 h post ICU admission could be useful as a predictive biomarker. Conversely, IL-6 remained unchanged in patients without sepsis throughout this study. However, IL-6 may not be a good biomarker for patients with non-septic illnesses.

NGAL is known as a biomarker of AKI; however, it is also an inflammatory biomarker because it is elevated in patients with sepsis [25]. Plasma NGAL levels reportedly have a role in differentiating septic AKI from non-septic AKI [26]. Kinetic analysis showed an elevation of NGAL levels only in non-survivors, which is consistent with the results of a previous report [27]. Although it is unclear whether this elevation was caused by progression of AKI or sepsis, it should be noted that a significant elevation within 6 h post ICU admission was associated with mortality.

HMGB1 is secreted by activated macrophages or released from necrotic or damaged cells and triggers local and systemic inflammation. Inflammatory responses induce injury to multiple organs [28]. Therefore, it is expected that HMGB1 will be a prognostic biomarker of sepsis and other non-infectious systemic inflammatory conditions. However, our study showed that HMGB1 levels were not different between survivors and non-survivors and were unchanged through 6 h post ICU admission among patients with and without sepsis. A previous study also reported that blood HMGB1 levels were unchanged and remained at an elevated level in patients with sepsis [12].

Given that each biomarker in this study had a modest predictivity with an AUC of 0.6–0.72, multiple biomarkers may be necessary for better identification of the responder to the treatment or predicting the prognosis because of complicated pathophysiology in severe illness, including sepsis [29]. Although the utility of lactate clearance in critically ill patients has been reported so far [6,8], this study found that the combination with lactate and HMGB1 may help stratify the risk of mortality better than lactate alone. The combination of biomarkers had a potential to predict ICU mortality better than one variable, although net reclassification improvement and integrated discrimination improvement did not provide the efficacy of the combination because of a small number in this study.

This study has several limitations. First, the sample size in this study was uncalculated and small, and thereby, the conclusion may not be applied to other clinical situations. Especially, the effect of the combination of biomarkers could not be proved because of the small number. The significance of this preliminary proof-of-concept study is that a comparison of inflammatory mediator kinetics in the same septic patients was conducted for the first time. Larger numbers of patients, including those with mild or moderate illness, should be evaluated to confirm the findings. Second, this is an observational study, which makes it unclear whether evaluation of inflammatory biomarker kinetics post ICU admission will improve the prognosis. Although evidence-based standardized treatment was provided to all patients, it is unclear if a specific treatment will change a biomarker’s kinetics. Further investigation is necessary for developing new therapeutic strategies based on biomarker kinetics.

## 5. Conclusions

In this preliminary study on critically ill patients in the ICU, different biomarker kinetics were observed. Reduction in lactate and elevation in NGAL levels were associated with survival; IL-6 level decrease was observed only in survivors with sepsis. HMGB1 levels were unchanged throughout the 6-h observation. Further studies will be needed to evaluate the efficacy of the combination of the inflammatory biomarkers with lactate and to determine treatment-specific biomarker kinetics that will guide therapeutic strategies against critical illnesses.

## Figures and Tables

**Table 1:** Baseline patient characteristics.

Characteristics	*n* = 30
Age	68 (51–76)
Male/Female	21/9
Hypertension	13 (43%)
Diabetes mellitus	9 (30%)
Admission type	
Medical	21 (70%)
Elective surgical	3 (10%)
Emergent surgical	6 (20%)
Sepsis	14 (47%)
- Blood culture positive	6 (43%)
- Infection site	
Gastrointestinal	8 (57%)
Pneumonia	2 (14%)
Urinary tract	1 (7%)
Meningitis	1 (7%)
Cholangitis	1 (7%)
Necrotizing fasciitis	1 (7%)
-Infecting organism	
Gram-positive cocci	4 (28%)
Gram-positive rod	3 (21%)
Gram-negative rod	4 (28%)
Charlson comorbidity score	1 (0–3)
APACHE II score	23 (18–26)
SAPS II score	54 (42–63)
SOFA score	9 (6–12)
Mechanical ventilation	22 (73%)
Acute kidney injury	25 (83%)
Dependent on catecholamine	12 (40%)
Duration of hospitalization (days)	30 (13–66)
Length of ICU stay	8 (4–15)
ICU mortality	14 (47%)
Survival days until ICU death (days)	8 (4–15)
In-hospital mortality	16 (53%)
Lactate at ICU admission (mmol/L)	6.4 (4.8–9.9)

APACHE, Acute Physiology and Chronic Health Evaluation; SAPS, simplified acute physiology score; SOFA, sequential organ failure assessment.

**Table 2:** The cutoff and area under the curve (AUC) of each biomarker for ICU mortality.

Biomarker	Cutoff	AUC (95% CI)
Lactate	0.70	0.73 (0.48–0.89)
IL-6	0.94	0.63 (0.40–0.81)
NGAL	1.00	0.72 (0.49–0.87)
HMGB1	1.20	0.58 (0.35–0.78)

## Supplementary Materials

Supplementary materials can be found at https://www.mdpi.com/2075-4418/10/7/454/s1.
