# Supplementary material for: Different Biomarker Kinetics in Critically Ill Patients with High Lactate Levels
Source: Diagnostics (Basel). 2020 Jul 4;10(7):454. doi: 10.3390/diagnostics10070454 (PMC7400035; doi:10.3390/diagnostics10070454)
Supplement: Supplementary file 1 [file diagnostics-10-00454-s001.pdf]

Different biomarker kinetics in critically ill patients with high lactate levels

Ryo Matsuura, M.D. Ph.D.[1], Yohei Komaru, M.D.[1], Yoshihisa Miyamoto, M.D.[1], Teruhiko Yoshida, M.D.[1], Kohei Yoshimoto, M.D.[2], Yoshifumi Hamasaki, M.D., Ph.D.[1], Masaomi Nangaku, M.D., Ph.D.[1] and Kent Doi, M.D., Ph.D.[2*]

Supplemental Table 1: Net reclassification improvement (NRI) and integrated discrimination improvement (IDI) for each model to predict ICU mortality.

	NRI	p-value	IDI	p-value
Overall				
Lac	reference		reference	
Lac + IL-6	-0.16 (-0.80 - 0.48)	0.63	0.006 (-0.021 - 0.033)	0.67
Lac + NGAL	0.18 (-0.44 - 0.79)	0.57	0.007 (-0.017 - 0.031)	0.57
Lac + HMGB1	0.05 (-0.55 - 0.65)	0.86	0.003 (-0.003 - 0.010)	0.27
Non-sepsis				
Lac	reference		reference	
Lac + IL-6	0.07 (-0.93 - 1.07)	0.89	0.033 (-0.050 - 0.116)	0.44
Lac + NGAL	0.51 (-0.37 - 1.39)	0.26	0.045 (-0.086 - 0.176)	0.5
Lac + HMGB1	0.30 (-0.75 - 1.33)	0.58	0.018 (-0.067 - 0.102)	0.68
Sepsis				
Lac	reference		reference	
Lac + IL-6	0.09 (-0.94 - 1.12)	0.87	0.012 (-0.062 - 0.087)	0.75
Lac + NGAL	0.60 (-0.33 - 1.53)	0.21	0.121 (-0.023 - 0.265)	0.1
Lac + HMGB1	0.13 (-0.88 - 1.14)	0.8	0.027 (-0.099 - 0.154)	0.67
